# Optimised Formulation of a New Sweet Apricot Kernel-Enriched Yoghurt: Assessment of Physicochemical, Sensory and Antioxidant Properties

**DOI:** 10.17113/ftb.62.02.24.8329

**Published:** 2024-06

**Authors:** Mohand Teffane, Hafid Boudries, Mostapha Bachir-bey, Ahcene Kadi, Younes Arroul, Abdeslem Taibi

**Affiliations:** 1 Laboratoire de Biomathématiques, Biophysique, Biochimie et Scientométrie (L3BS), Faculté des Sciences de la Nature et de la Vie, Université de Bejaia, 06000 Bejaia, Algeria; 2 Laboratoire de Biochimie Appliquée, Faculté des Sciences de la Nature et de la Vie, Université de Bejaia, 06000 Bejaia, Algeria

**Keywords:** yoghurt, sweet apricot kernel, antioxidant activity, formulation optimisation, physicochemical properties, sensory properties

## Abstract

**Research background:**

The addition of sweet apricot kernel powder, a by-product of apricot processing, to yoghurt appears to be particularly interesting option for the innovation of new food products. This study focuses on the formulation of a novel yoghurt enriched with sweet apricot kernel powder, sugar and milk powder.

**Experimental approach:**

Different yoghurts were prepared by mixing sweet apricot kernel powder, sugar and milk powder as ingredients based on the simplex-centroid mixture design. The optimisation process took into account the physicochemical, antioxidant and sensory properties of the yoghurt.

**Results and conclusions:**

The results showed that the optimum values of sugar, milk powder and apricot kernel powder were 3.07, 2.16 and 2.77 %, respectively. The physicochemical assays showed that the addition of apricot kernel powder led to a significant increase in total phenolic content, antioxidant activity, syneresis, viscosity and acidity. The addition of sugar and milk powder also had a significant effect on the taste, texture and consistency of the yoghurt. Moreover, the enrichment of the product with apricot kernel powder significantly influenced the colour, odour, taste, texture and consistency. In conclusion, the optimised yoghurt enriched with apricot kernel had an interesting phenolic content and antioxidant properties with sensory acceptability, while reducing the amount of sugar and milk powder. This confirms the potential of using sweet apricot kernels as an ingredient in yoghurt production.

**Novelty and scientific contribution:**

The use of a simplex-centroid mixture design to optimise a new yoghurt formulation enriched with sweet apricot kernels shows significant improvements in total phenolic content, antioxidant activity and sensory acceptability. In addition, less sugar and milk powder is needed. The addition of sweet apricot kernels to yoghurt is therefore a new approach to improving its nutritional value and sensory appeal.

## INTRODUCTION

Apricot tree (*Prunus armeniaca* L.) is grown in 68 countries worldwide and produces around 3.84 million tonnes of apricot fruit. Apricots are mostly cultivated in the Mediterranean region (*1*–*3*). The processing of apricot fruit produces significant quantities of kernels, which are often discarded by the industry, resulting in the loss of potentially valuable resources and environmental pollution (*4*, *5*). These kernels contain many beneficial compounds such as unsaturated fatty acids, proteins, bioactive components such as phenolic acids, flavonoids, essential minerals and vitamins (*6*). Therefore, apricot kernels are widely used in the cosmetic, pharmaceutical and food industries (*7*–*9*).

As far as we know, the use of bitter apricot kernels in food production is severely limited due to the presence of amygdalin. However, the sweet and detoxified kernels have been used for the production of cookies (*9*–*11*) and dairy products (*12*–*14*).

Dairy products are widely consumed foods worldwide and have seen a substantial increase in consumption in recent years (*15*). Among the popular dairy products, yoghurt is highly valued by consumers. It has been known for centuries for its therapeutic benefits (*16*). Yoghurt consumption has been reported to help relieve diarrhoea, shorten colonic transit time, boost immunity and contribute to lower serum cholesterol levels (*16*).

Continuing the work on apricot kernel utilisation, food waste management is becoming an issue from both an environmental and economic perspective, as it not only reduces pollution but also offers new opportunities for economic development. As part of the innovation of new food products to meet consumer demand for natural, nutritious and health-promoting products, dairy products also include a wide range of products. The incorporation of sweet apricot kernel powder into yoghurt is highly desirable. However, research has not yet been conducted to optimise the amounts of sweet apricot kernels with sugar and milk powder and to determine their effects on the physicochemical, antioxidant and sensory properties of yoghurt. This optimisation is necessary to reduce the quantity of added ingredients and to determine their effects and interactions in yoghurt.

The aim of this study therefore is to optimise the combination of three ingredients in yoghurt using simplex-centroid mixture design. The main objective is to develop a yoghurt with low sugar content and high antioxidant content that not only meets quality standards but also satisfies sensory preferences of consumers. To achieve this, different physicochemical properties such as total phenolic content (TPC), 2,2-diphenyl-1-picrylhydrazyl (DPPH) free radical scavenging activity, 2,2'-azino-bis(3-ethylbenzthiazoline-6-sulfonic acid) (ABTS) radical scavenging activity, syneresis, viscosity, pH and acidity, as well as sensory properties including texture, taste and appearance are carefully evaluated.

## MATERIALS AND METHODS

### Raw materials

Milk, sugar and milk powder were purchased from a market in the city of Bejaia, Algeria. The sweet apricot kernels were kindly provided by the apricot processing industry of N'gaous in the department of Batna, Algeria, in July 2021. They were ground and sieved to a diameter of less than 1 mm.

### Physicochemical and antioxidant properties of sweet apricot kernel powder

The physicochemical and antioxidant properties of sweet apricot kernel powder were analysed using standard protocols described in various studies. Moisture content was measured by drying 5 g samples at 105 °C until a constant mass was reached using a ventilated oven (Memmert GmbH + Co.KG, Schwabach, Germany) (*5*). Ash content was determined by incinerating 5 g of sample in a muffle furnace at 600 °C for 4 h (LE 2/11/R6; Nabertherm GmbH, Lilienthal, Germany) (*17*). Lipids from the sweet apricot kernel powder were extracted by ultrasound-assisted extraction (2510E-DTH; Branson Ultrasonics Corporation, Danbury, CT, USA) with *n*-hexane at 50 °C for 40 min. After filtration and evaporation of the solvent, the oil yield was calculated (*18*). Total protein was determined using the Bradford method (*19*) by mixing 200 μL of the extract, obtained from 1 g of powder with 20 mL of 70 % ethanol for 24 h, with 2.5 mL of Bradford reagent and measuring the absorbance at 596 nm. The carbohydrate content was determined by extracting 0.5 g of the sample with 20 mL of 80 % ethanol, then incubating at 95 °C and centrifuging at 4193×*g* for 15 min (Digicen 21R centrifuge; Orto Alresa, Madrid, Spain). The supernatant (1 mL) was then mixed with 0.5 mL of 5 % phenol and 2.5 mL of sulfuric acid, and the absorbance was measured at 490 nm after a 20-minute incubation at 80 °C (*20*). The total hydrogen cyanide content was estimated by immersing 10 g of apricot kernel powder in a phosphoric acid solution, distilling the mixture and titrating the distillate with AgNO_3_ (*21*). Polyphenols were extracted by shaking 0.5 g of apricot kernel powder with 20 mL of 50 % acetone in a magnetic stirrer (AM4; VELP Scientifica, Usmate, Italy) (*22*). Total phenolic content (TPC) was measured using the Folin-Ciocalteu method (*23*), in which the extracts were reacted with the Folin-Ciocalteu reagent and sodium carbonate and the absorbance was measured at 765 nm using spectrophotometer (UV mini1240; Shimadzu, Suzhou Jiangsu, PR China). Total flavonoid content (TFC) was determined by mixing 1 mL of extract with 1 mL of 2 % aluminium chloride solution and measuring the absorbance at 430 nm (*24*). Tannin content (TC) was determined by mixing 50 µL of extract with 1.5 mL of 4 % methanol solution of vanillin and HCl and measuring the absorbance at 500 nm (*25*). The antioxidant activities (DPPH and ABTS radical scavenging) were determined according to the methods of Alam *et al*. (*26*) and Pérez-Chabela *et al*. (*27*), respectively. Samples were mixed with DPPH or ABTS^+^ solutions and the absorbance was measured after 30 min at 517 nm for DPPH radical scavenging activity or after 6 min at 734 nm for ABTS radical scavenging activity, with results expressed as percentage of inhibition.

### Yoghurt preparation and experimental design

The yoghurts were processed according to the method described by Felfoul *et al.* (*28*). The milk mixture with sugar, milk powder and sweet apricot kernel powder was pasteurised at 85 °C for 30 min and then cooled to 45 °C. It was then filled into 180-mL plastic bottles and incubated at 45 °C for 6 h with 0.01 g/L of lactic acid bacteria (*Streptococcus thermophilus* and *Lactobacillus delbrueckii* ssp. *bulgaricus*). After incubation, the yoghurts were cooled at 4 °C for 48 h and mixed to disperse the apricot kernel husk for homogenisation. They were then stored for further analysis. All experiments were done in triplicate.

In order to obtain an acceptable yoghurt product with optimal physicochemical, antioxidant and sensory properties, a simplex-centroid mixture design with ten formulations was created. The design aimed to optimise and evaluate the effects of three ingredients: sugar (X_1_), milk powder (X_2_) and sweet apricot kernel powder (X_3_). The total mass fraction of added ingredients in each formulation was fixed at 8 %, and the proportions of the components were expressed as fractions of the mixture, where the sum of the ingredients (X_1_+X_2_+X_3_=1) represents coded value. The experimental design included ten combinations of the three factors at different levels, while the 11th formulation (K) served as a negative control without any added ingredients (Table 1).

**Table 1:** Coded and real values (in brackets) of the yoghurt formulations with the addition of sugar, milk powder and sweet apricot kernel powder

		*w*(ingredient)/(g/100 g)	
Formulation	X_1_	X_2_	X_3_
A	1.000 (8.00)	0.000 (0.00)	0.000 (0.00)
B	0.167 (1.33)	0.167 (1.33)	0.667 (5.33)
C	0.667 (5.33)	0.167 (1.33)	0.667 (1.33)
D	0.500 (4.00)	0.500 (4.00)	0.000 (0.00)
E	0.000 (0.00)	1.000 (8.00)	0.000 (0.00)
F	0.333 (2.67)	0.333 (2.67)	0.333 (2.67)
G	0.167 (1.33)	0.667 (5.33)	0.167 (1.33)
H	0.000 (0.00)	0.000 (0.00)	1.000 (8.00)
I	0.000 (0.00)	0.500 (4.00)	0.500 (4.00)
J	0.500 (4.00)	0.000 (0.00)	0.500 (4.00)
K	0.000 (0.00)	0.000 (0.00)	0.000 (0.00)

X_1_=sugar, X_2_=milk powder, X_3_=sweet apricot kernel powder

The statistical analysis of the responses for each yoghurt formulation was performed using the JMP software, v. 10.0.0 (*29*). The analysis of variance (ANOVA) was used to determine the significance of the independent variables and their interactions, as well as the statistical significance of the regression coefficients and the adequacy of the model fit. The level of significance for each predicted response was set at α=0.05. The polynomial model is represented by the following equation:


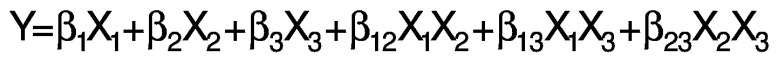
 /1/

where Y is the response (pH, acidity, viscosity, syneresis, TPC, DPPH, ABTS, taste, texture, consistency colour, odour and overall acceptability); β_1_, β_2_, β_3_, β_12_, β_13_ and β_23_ represent the coefficients of the factors and their interactions; X represents the concentration of each ingredient: X_1_ for sugar, X_2_ for milk powder and X_3_ for sweet apricot kernel powder.

### Physicochemical analysis and antioxidant properties of yoghurt

The pH and syneresis were determined using the method of Pachekrepapol *et al.* (*30*). After 24 h of storage at 4 °C, the pH was evaluated using a Sension+PH3 pH-meter (Hach, Loveland, CO, USA). The pH was measured directly on yoghurt samples at about 14 °C.

For syneresis, 5 g of yoghurt were centrifuged (Digicen 21R centrifuge; Orto Alresa, Madrid, Spain) at 2000×*g* and 4 °C for 20 min. The degree of syneresis (%) was calculated using the following equation:


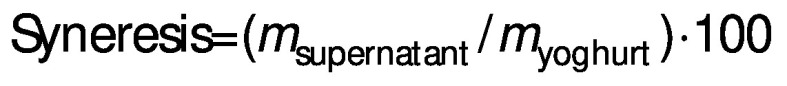
 /2/

The titratable acidity of the formulated yoghurts was measured according to the method described by Pérez-Chabela *et al.* (*27*). Yoghurt samples (10 g) were mixed with 40 mL of distilled water and 3 drops of phenolphthalein were added and then titrated with 0.1 M NaOH until a light pink colour appeared.

The viscosity of the formulated yoghurts was measured according to Felfoul *et al.* (*28*). The viscosity was determined using a viscometer (SNB-1; Precision Instruments, Shanghai, PR China) with a spindle no. 4 at 60 rpm for 30 s and expressed in Pa·s.

### Phenolic compound extraction and antioxidant activity

The phenolic compounds were extracted according to the method described by Pérez-Chabela *et al.* (*27*). Briefly, 1 g of the sample was macerated in 40 mL of *V*(acetone):*V*(water)=1:2 for 30 min at room temperature (22 °C) using a magnetic stirrer (AM4; VELP Scientifica). The resulting crude extracts were then filtered and centrifuged at 25975×*g* for 10 min.

The total phenolic content (TPC) was determined according to the method described by Goli *et al.* (*31*). In brief, 0.2 mL of each extract was mixed with 1 mL of Folin-Ciocalteu reagent (diluted 1:10 with distilled water), followed by the addition of 0.8 mL of 7.5 % sodium carbonate solution. The absorbance was measured after 30 min of incubation at 765 nm using a spectrophotometer (UV mini1240; Shimadzu, Suzhou Jiangsu, PR China). The TPC was expressed in mg of gallic acid equivalents (GAE) per 100 g of yoghurt.

The DPPH free radical scavenging was tested according to the method described by Alam *et al.* (*26*). A volume of 1 mL of DPPH stock solution (0.5 mM) was diluted with methanol to obtain the absorbance of 0.80±0.01 at 517 nm. Then, 0.9 mL of the diluted DPPH solution was added to 0.1 mL of the extract. After incubation for 30 min, the absorbance was measured at 517 nm. The percentage of inhibition of the DPPH free radical scavenging was calculated using the following formula:


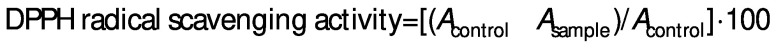
 /3/

The antioxidant potential of the ABTS^+^ radical scavenging was tested according to Pérez-Chabela *et al.* (*27*). The ABTS^+^ radical was generated by reacting 7 mM ABTS and 2.45 mM potassium persulfate after incubation for 12 h. The ABTS^+^ solution was then diluted with distilled water until an absorbance of 0.70±0.02 at 734 nm was achieved. Then, 0.1 mL of the extract was mixed with 0.9 mL of ABTS^+^ working solution and the absorbance was measured after 6 min at 734 nm. The percentage of ABTS free radical scavenging was calculated using the following formula:


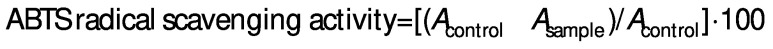
 /4/

### Sensory evaluation of the yoghurt

The sensory properties were evaluated according to the method described by Felfoul *et al.* (*28*). The yoghurt samples were taken out of the refrigerator and allowed to stand at room temperature for about 1 min. The serving temperature for the samples was approx. 10 to 12 °C. Each yoghurt sample was presented in a 35-mL plastic cup with a lid and each cup was labelled with an alphabetical code. The order of presentation of the samples was randomised, with seven samples presented in the first session and four samples presented in the second tasting session.

For the evaluation, a panel of 18 people was invited to evaluate the colour, texture, consistency, odour and taste of the yoghurt samples. The panel consisted of 14 trained female experts and 4 untrained male participants, aged between 25 and 48. The overall acceptability of the yoghurts was evaluated using a 9-point hedonic scale, where 1=extremely unpleasant, 2=very unpleasant, 3=moderately unpleasant, 4=slightly unpleasant, 5=neither pleasant nor unpleasant, 6=slightly pleasant, 7=moderately pleasant, 8=very pleasant and 9=extremely pleasant.

### Statistical analyses

The mean value±standard deviation of the tests were given. To determine significant differences between the mean values at a significance level of 5 %, one-way analysis of variance (ANOVA) with the Fisher’s LSD test was used to compare the mean values between the physicochemical and antioxidant properties. For the sensory characteristics, the Kruskal-Wallis and Conover-Iman tests were used to compare the mean values. All mathematical calculations and statistical analyses were conducted using Statistica, v. 13.3.0 (*32*), the demo versions of the MS Office XLSTAT, MS Office demo version 2014.5.034 (*33*) and JMP, v. 10.0.0 (*29*) software.

## RESULTS AND DISCUSSION

### The results of physicochemical and antioxidant analyses of sweet apricot kernel powder

The physicochemical properties of the apricot kernel powder used in the yoghurt formulations are shown in Table 2. The moisture, ash, protein, lipid and carbohydrate mass fractions were estimated to be (8.8±0.9), (3.5±0.1), (1.3±0.2), (56.2±2.0) and (8.8±0.9) %, respectively. These results were in agreement with those of Fayed (*34*) and Arafa (*35*). These authors reported that the moisture mass fraction of apricot kernels ranged from 3.25 to 13.56 %, depending on the variety. Femenia *et al.* (*36*) observed a mass fraction of moisture from 5.4 to 6.7 %, a carbohydrate 5 to 20 % and lipid 40 to 56 %. Similarly, Hayta and Alpaslan (*37*) reported that the mass fraction of carbohydrate in apricot kernels ranged from 17 to 27.9 %, protein from 14.1 to 45.3 % and lipid from 27.7 to 66.7 %. In addition, Mohamed *et al.* (*38*) reported a mass fraction of ash 2.2 %, a carbohydrate of 31.56 %, protein of 22.6 % and a lipid of 42.8 % in apricot kernels.

**Table 2:** Physicochemical and antioxidant properties of the sweet apricot kernel powder used in the yoghurt formulations

Physicochemical property	Amount
*w*(moisture)/%	8.8±0.9
*w*(ash)/%	3.5±0.1
*w*(carbohydrate)/%	8.8±0.9
*w*(protein)/%	1.3±0.2
*w*(lipid)/%	56.2±2.0
TPC as *w*(GAE)/(mg/100 g)	271.9±3.7
TFC as *w*(GAE)/(mg/100 g)	1.8±0.2
TC as *w*(GAE)/(mg/100 g)	110.0±14.8
DPPH/%	34.0±2.8
ABTS/%	74.2±1.9
*w*(HCN)/(mg/100 g)	57.6±6.2

TPC=total phenolic content, TFC=total flavonoid content, TC=tannin content, GAE=gallic acid equivalent, DPPH=2,2-diphenyl-1-picrylhydrazyl free radical scavenging activity, ABTS=2,2'-azino-bis(3-ethylbenzthiazoline-6-sulphonic acid) radical scavenging activity

The apricot kernel powder used in the yoghurt formulations had a total phenolic content, expressed as gallic acid equivalents (GAE), of (271.9±3.7) mg/100 g, a total flavonoid content, expressed as GAE, of (1.8±0.2) mg/100 g and a tannin content, expressed as GAE, of (110.0±14.8) mg/100 g. The antioxidant activity measured with ABTS was (74.2±1.9) % and with DPPH (34.0±2.8) % (Table 2). Similarly, Tanwar *et al.* (*21*) found a high content of tannins compared to flavonoids in apricot kernels, with a total phenolic, tannin and flavonoid contents of (183.1±6.5), (159.7±8.2) and (14.8±2.0) mg/100 g, respectively. In addition, Rampáčková *et al.* (*39*) reported that the total phenolic content, expressed as GAE on dry mass basis, in apricot kernels ranged from 63.5 to 1277.3 mg/100 g, and the total flavonoid from 0 to 153.1 mg/100 g, with antioxidant activity, expressed as Trolox equivalents, ranging from 0.483 to 2.348 mg/100 g. Moreover, Mohamed *et al.* (*38*) determined the total phenolic content of apricot kernels to be 7.7 mg/100 g and the flavonoid content to be 4.03 mg/100 g. It is important to note that the differences in phenolic content of kernels can be attributed to several factors such as genetic diversity, geographical location, growth conditions, harvest time, soil composition, choice of extraction solvent, chemical composition and the used method of analysis (*23*, *40*).

The estimated hydrogen cyanide mass fraction of sweet apricot kernels used in this study was (57.6±6.2) mg/100 g (Table 2). Recently, Pawar and Nema (*41*) reported that the HCN content of apricot kernels varies with cultivar/variety, altitude of the region and maturity. Based on the amount of HCN contained in the apricot kernels, they distinguished between sweet and bitter kernels. The bitter apricot kernel contains a large amount of HCN, *i.e.* 220.55 to 317.7 mg/100 g compared to sweet apricot kernels, *i.e.* 30.20 to 79.20 mg/100 g (*41*). According to Tanwar *et al.* (*21*), the mass fraction of HCN in wild apricot kernels is estimated to be (136.85±2.67) mg/100 g. Generally, the hydrogen cyanide content in apricot kernels varies widely, ranging from 12.2 to 409 mg/100 g, with a mean content of 29.20 mg/100 g (*42*).

### Physicochemical and antioxidant properties of yoghurt

The physicochemical properties of the yoghurt formulations are shown in Table 3. The pH values varied between 4.47 and 4.70. The formulations labelled E (0 % sugar, 8 % milk powder and 0 % sweet apricot kernel powder) and H (0 % sugar, 0 % milk powder and 8 % sweet apricot kernel powder) are yoghurts without sugar and had the pH higher than 4.6. The acidity values of all formulations were between 7.6 and 12.3 g/L. The lowest and highest acidity values were observed in yoghurts A (8 % sugar, 0 % milk powder and 0 % sweet apricot kernel powder) and E (0 % sugar, 8 % milk powder and 0 % sweet apricot kernel powder), respectively. According to Zahan *et al.* (*43*), a total of 12 set-type yoghurt samples with four sugar mass fractions (0, 4, 5 and 12 %) were used, and the plain yoghurt had an average acidity (0.9 %) and pH (4.45). Additionally, Elkot *et al.* (*14*) reported that titratable acidity and pH in yoghurt samples supplemented with detoxified apricot kernels were between 0.61 and 0.75 % and 4.55 and 4.65, respectively. Furthermore, Karnopp *et al.* (*44*) reported pH values of 4.08–4.29 and acidity values of 0.64–0.77 % in yoghurt made with grape skin flour, oligofructose and purple grape juice.

**Table 3:** Physicochemical and antioxidant properties of the yoghurt formulations

Formulation	pH	*γ*(acidity)/(g/L)	*η*/(Pa·s)	Syneresis/%	TPC as *w*(GAE)/(mg/100 g)	DPPH/%	ABTS/%
A	(4.62±0.03)^abc^	(7.6±0.8)^f^	(6.6±0.4)^abc^	(62.2±0.6)^ab^	(78.1±9.8)^ef^	(22.1±3.1)^bcd^	(42.9±1.8)^e^
B	(4.53±0.01)^bcd^	(9.9±0.1)^bc^	(4.5±1.2)^cd^	(56.5±1.8)^def^	(201±11)^a^	(24.3±1.2)^bc^	(60.8±5.2)^c^
C	(4.53±0.01)^bcd^	(9.05±0.07)^cde^	(5.8±1.7)^abc^	(57.67±0.00)^de^	(102±16)^d^	(21.3±0.1)^cd^	(39.1±2.6)^e^
D	(4.65±0.01)^ab^	(9.05±0.07)^cde^	(6.9±0.5)^ab^	(53.92±0.00)^f^	(67.1±13.8)^fj^	(21.2±1.8)^cd^	(42.3±1.7)^e^
E	(4.70±0.01)^a^	(12.3±0.7)^a^	(7.2±0.4)^a^	(41.3±1.0)^h^	(95.9±1.6)^de^	(21.2±1.0)^cd^	(42.5±5.6)^e^
F	(4.6±0.2)^bcd^	(9.2±0.4)^cd^	(5.6±0.9)^abc^	(55.0±0.2)^ef^	(141.6±0.4)^c^	(24.0±1.6)^bc^	(52.1±0.7)^d^
G	(4.62±0.05)^abc^	(10.8±0.3)^b^	(6.3±1.9)^abc^	(49.2±2.8) ^j^	(103.9±9.4)^d^	(21.0±0.5)^cd^	(55.0±0.7)^cd^
H	(4.71±0.02)^a^	(8.3±0.2)^ef^	(5.1±1.1)^c^	(61.6±1.2)^abc^	(200.0±2.4)^a^	(40.7±3.0)^a^	(79.7±1.8)^a^
I	(4.51±0.09)^cd^	(10.6±0.1)^b^	(2.9±0.2)^de^	(58.3±3.6)^cde^	(139.6±1.6)^c^	(26.1±1.2)^b^	(68.7±0.72)^b^
J	(4.47±0.01)^d^	(9.00±0.00)^cde^	(5.2±1.3)^bc^	(59.2±0.2)^bcd^	(167.5±2.0)^b^	(23.1±3.4)^bcd^	(55.6±1.2)^cd^
K	(4.53±0.06)^bcd^	(8.5±0.7)^def^	(2.2±0.2)^e^	(63.0±1.0)^a^	(52.8±2.4)^j^	(19.2±0.3)^d^	(37.6±1.4)^e^

Results with different letters in superscript in the same row are statistically significantly different (p<0.05, a>b>c>d). Samples were compared using one-way analysis of variance (ANOVA) with the Fisher’s LSD test. GAE=gallic acid equivalent, DPPH=2,2-diphenyl-1-picrylhydrazyl free radical scavenging activity, ABTS=2,2'-azino-bis(3-ethylbenzthiazoline-6-sulphonic acid) radical scavenging activity

The acidity and pH observed in the present study emphasise the importance of milk powder and added sugar in achieving the desired pH and acidity. Milk powder and sugar play an important role in yoghurt production, as they act as substrates for the metabolism of the lactic acid bacteria present in the yoghurt and lead to the formation of lactic acid, which acidifies the medium.

Regarding viscosity and syneresis, it was found that formula E, with the highest viscosity (7.2 Pa·s), had the lowest syneresis rate (41.3 %), which was attributed to the effect of the milk powder (Table 3). The milk powder probably contains hydrophilic proteins that retain water, which leads to an increase in viscosity. In agreement with this, Soukoulis *et al.* (*45*) observed that skimmed milk powder improves the textural quality and reduces the susceptibility of yoghurts to syneresis. Moreover, Celik *et al.* (*46*) reported that lower viscosity and higher syneresis are characteristics of fruit yoghurt. They explained that the addition of concentrated fruits reduces the water retention capacity of the proteins, which dilutes the protein content in the milk base, decreases viscosity and increases syneresis in fruit-flavoured yoghurt.

Formulations B (1.33 % sugar, 1.33 % milk powder and 5.33 % sweet apricot kernel powder) and H (0 % sugar, 0 % milk powder and 8 % sweet apricot kernel powder) had higher total phenolic content (TPC) and mean values for ABTS and DPPH radical scavenging activities, suggesting that apricot kernels are a good alternative for increasing the *in vitro* antioxidant activity of yoghurt (Table 3). However, these formulations were not the most preferred by the panellists during sensory evaluation.

### Sensory properties of yoghurt

The sensory evaluation scores in Table 4 shows that no significant difference in colour or odour was noticed between the 11 different yoghurt formulations, while other parameters such as taste, texture and consistency were significantly influenced by the addition of sweet apricot kernel powder, sugar and milk powder (p*<*0.05). Formulations E (0 % sugar, 8 % milk powder and 0 % sweet apricot kernel powder), H (0 % sugar, 0 % milk powder and 8 % sweet apricot kernel powder) and the control yoghurt K (0 % sugar, 0 % milk powder and 0 % sweet apricot kernel powder) achieved the lowest mean scores for overall acceptability, namely 4.61, 4.83 and 3.67, respectively. On the other hand, formulations C (5.33 % sugar, 1.33 % milk powder and 1.33 % sweet apricot kernel powder) and D (4 % sugar, 4 % milk powder and 0 % sweet apricot kernel powder) had the highest mean scores of overall acceptability, namely 6.06 and 6, respectively. According to Zahan *et al.* (*43*), a higher concentration of sugar should lead to an increase in preference. However, the acceptability of sweet taste varies from consumer to consumer. Furthermore, the differences in sensory evaluation of yoghurt samples generally depend on the type of milk, ferment and production process.

**Table 4:** Sensory evaluation of the yoghurt formulations

Formulation	Colour	Odour	Taste	Texture	Consistency	Overall acceptability
A	(5.8±1.5)^a^	(5.2±1.2)^a^	(5.4±1.3)^abc^	(4.9±1.3)^bc^	(4.6±1.5)^b^	(5.3±1.2)^abc^
B	(5.9±1.76)^a^	(6.0±1.4)^a^	(5.6±1.8)^abc^	(5.7±1.7)^abc^	(5.4±1.7)^ab^	(5.7±1.2)^ab^
C	(6.4±1.24)^a^	(5.5±1.1)^a^	(6.1±2.0)^ab^	(5.8±1.6)^ab^	(5.7±1.7)^ab^	(6.1±1.9)^a^
D	(6.1±1.15)^a^	(5.3±1.2)^a^	(6.1±1.3)^ab^	(5.9±1.2)^ab^	(6.1±1.3)^a^	(6.0±1.1)^a^
E	(5.8±1.67)^a^	(5.1±1.8)^a^	(4.8±1.9)^c^	(5.5±1.8)^abc^	(6.5±1.7)^a^	(4.6±1.6)^cd^
F	(6.4±0.86)^a^	(5.8±1.3)^a^	(6.1±1.7)^ab^	(5.8±1.3)^ab^	(5.9±1.2)^a^	(5.8±1.8)^ab^
G	(6.1±1.49)^a^	(5.3±1.3)^a^	(5.3±0.9)^abc^	(6.1±1.0)^a^	(6.0±1.1)^a^	(5.4±1.4)^abc^
H	(5.5±2.33)^a^	(5.3±2.1)^a^	(4.9±1.6)^c^	(4.9±2.2)^abc^	(5.2±1.8)^ab^	(4.8±1.4)^bcd^
I	(6.2±1.42)^a^	(5.9±1.5)^a^	(4.9±1.8)^bc^	(5.9±1.7)^ab^	(6.1±1.7)^a^	(5.5±1.4)^abc^
J	(6.2±1.21)^a^	(6.0±1.1)^a^	(6.2±1.6)^a^	(5.8±1.4)^abc^	(5.4±1.9)^ab^	(5.8±1.8)^ab^
K	(5.6±1.70)^a^	(5.0±1.8)^a^	(4.3±2.1)^c^	(4.7±1.7)^c^	(4.4±2.0)^b^	(3.7±2.1)^d^

Results with different letters in superscript in the same row are statistically significantly different (p<0.05, a>b>c>d). Pairwise comparisons of samples were conducted using the Conover-Iman test. A 9-point hedonic scale ranging from 1 (extremely unpleasant) to 9 (extremely pleasant) was used

According to Cheng *et al*. (*47*), certain naturally occurring volatile organic compounds (VOCs) in milk and those produced during lactic acid fermentation contribute to the variety of aromas and flavours perceived by consumers. Kilcawley *et al.* (*48*) further reported that short-chain carboxylic acids, which are typically the most abundant chemical class of VOCs in many dairy products, are the main components responsible for sour taste and, in some cases, rancidity. These acids come from a variety of sources and pathways, including lipolysis, carbohydrate metabolism or amino acid metabolism, depending on the specific short-chain carboxylic acid. Moreover, volatile compounds such as aldehydes, ketones, sulfur compounds, terpenes, *etc*. present in dairy products such as milk and derived from the degradation of lactose, citrate, milk lipids and milk protein or produced after fermentation (*e.g*. yoghurt), such as acetaldehyde, also contribute to different sensory evaluation of yoghurt samples (*47*).

### Model fitting and regression analysis

The ANOVA results for the model fitted for the physicochemical and antioxidant properties as well as the sensory evaluation of the yoghurt are shown in Table 5. All mathematical models were statistically significant (p*<*0.05) and the coefficients of determination (R^2^) for pH (R^2^=0.960), acidity (R^2^=0.966), viscosity (R^2^=0.912), syneresis (R^2^=0.961), TPC (R^2^=0.962), DPPH (R^2^=0.938), ABTS (R^2^=0.939), colour (R^2^=0.892), odour (R^2^=0.914), taste (R^2^=0.977), texture (R^2^=0.954), consistency (R^2^=0.933) and overall acceptance (R^2^=0.951) were high. This indicates that the models can explain the effects of the factors well and the equations can be used to predict the responses.

**Table 5:** ANOVA results for the model fitted for physicochemical, antioxidant properties and sensorial evaluation of the yoghurt

Term	Coeff.	p-value	Coeff.	p-value	Coeff.	p-value	Coeff.	p-value	Coeff.	p-value	Coeff.	p-value	Coeff.	p-value
	pH		Acidity		Viscosity		Syneresis		TPC		DPPH		ABTS	
X_1_	4.611	<0.0001*	7.672	<0.0001*	6.371	0.0003*	62.639	<0.0001*	73.701	0.0057*	22.343	0.0005*	41.894	0.0009*
X_2_	4.704	<0.0001*	12.277	<0.0001*	7.233	0.0002*	41.358	<0.0001*	92.923	0.0024*	21.267	0.0006*	44.608	0.0007*
X_3_	4.700	<0.0001*	8.390	<0.0001*	5.006	0.0008*	61.315	<0.0001*	204.984	0.0001*	39.630	<0.0001*	78.863	<0.0001*
X_1_ˣX_2_	0.016	0.893	-3.563	0.099	2.047	0.459	3.001	0.733	-59.722	0.395	0.047	0.9964	-8.562	0.711
X_1_ˣX_3_	-0.712	0.003*	4.504	0.054	-1.099	0.683	-16.997	0.107	149.440	0.076	-33.052	0.0279*	-35.869	0.171
X_2_ˣX_3_	-0.707	0.003*	13.13	0.474	-10.701	0.013*	20.462	0.067	4.925	0.941	-20.051	0.1099	23.930	0.328
R^2^	0.960		0.966		0.912		0.961		0.962		0.938		0.939	
R^2^_adj_	0.908		0.924		0.804		0.913		0.914		0.862		0.864	
Model		0.007*		0.005*		0.030*		0.006*		0.006*		0.016*		0.015*
Term	Colour		Odour		Taste		Texture		Consistency		Overall acceptability			
X_1_	5.873	<0.0001*	5.166	<0.0001*	5.407	<0.0001*	4.918	<0.0001*	4.739	<0.0001*	5.336	<0.0001*		
X_2_	5.79	<0.0001*	5.065	<0.0001*	4.768	<0.0001*	5.564	<0.0001*	6.374	<0.0001*	4.570	<0.0001*		
X_3_	5.451	<0.0001*	5.391	<0.0001*	4.910	<0.0001*	4.942	<0.0001*	5.186	<0.0001*	4.872	<0.0001*		
X_1_ˣX_2_	1.324	0.103	0.230	0.753	3.710	0.0024*	2.466	0.013*	1.922	0.098	3.866	0.005*		
X_1_ˣX_3_	2.332	0.021*	2.845	0.014*	4.164	0.0015*	3.246	0.005*	1.693	0.131	2.691	0.019*		
X_2_ˣX_3_	2.074	0.030*	2.407	0.024*	0.321	0.5839	2.338	0.015*	0.529	0.585	2.598	0.021*		
R^2^	0.892		0.914		0.977		0.954		0.933		0.951			
R^2^_adj_	0.757		0.807		0.949		0.897		0.850		0.890			
Model		0.045*		0.030*		0.002*		0.008*		0.018*		0.010*		

Coeff.=regression coefficient, X_1_=sugar, X_2_=milk powder, X_3_=sweet apricot kernel powder, *significant coefficient (p*<*0.05), R^2^=coefficient of determination,

R^2^_adj_=adjusted coefficient of determination, DPPH=2,2-diphenyl-1-picrylhydrazyl free radical scavenging activity, ABTS=2,2'-azino-bis(3-ethylbenzthiazoline-6-sulphonic acid) radical scavenging activity

Table 5 and Table 6 show the effects of sugar (X_1_), milk powder (X_2_) and sweet apricot kernel powder (X_3_) on the physicochemical, antioxidant and sensory properties of yoghurt. The linear effects were found to be significant (p<0.05). However, not all interactions between these ingredients were significant. The interactions of sugar and sweet apricot kernel powder (X_1_×X_3_) and milk powder and sweet apricot kernel powder (X_2_×X_3_) had a negative effect on the pH and DPPH radical scavenging activity and a positive effect on the texture, odour and colour characteristics. The interaction of milk powder and sweet apricot kernel powder (X_2_×X_3_) had a negative effect on the viscosity. The taste characteristic was positively influenced by the interaction of sugar and sweet apricot kernel powder (X_1_×X_3_). Moreover, the overall acceptability of the product was positively influenced by all ingredients and their interactions in the final product.

**Table 6:** The models developed for physicochemical, antioxidant and sensory properties of enriched yoghurt

Parameter	Model
pH	4.611X_1_+4.704X_2_+4.700X_3_-0.712X_1_X_3_-0.707X_2_X_3_
Acidity	7.672X_1_+12.277X_2_+8.390X_3_
Viscosity	6.371X_1_+7.233X_2_+5.006X_3_-10.701X_2_X_3_
Syneresis	62.639X_1_+41.358X_2_+61.315X_3_
TPC	73.701X_1_+92.923X_2_+204.984X_3_
DPPH	22.345X_1_+21.267X_2_+39.630X_3_-33.052X_1_X_3_
ABTS	41.894X_1_+44.608X_2_+78.863X_3_
Colour	5.873X_1_+5.788X_2_+5.451X_3_+2.332X_1_X_3_+2.074X_2_X_3_
Odour	5.166X_1_+5.065X_2_+5.391X_3_+2.845X_1_X_3_+2.407X_2_X_3_
Taste	5.407X_1_+4.768X_2_+4.910X_3_+3.710X_1_X_2_+4.164X_1_X_3_
Texture	4.918X_1_+5.564X_2_+4.942X_3_+2.466X_1_X_2_+3.246X_1_X_3_+2.338X_2_X_3_
Consistency	4.739X_1_+6.373X_2_+5.186X_3_
Overall acceptability	5.336X_1_+4.570X_2_+4.872X_3_+3.866X_1_X_2_+2.691X_1_X_3_+2.598X_2_X_3_

X_1_=sugar, X_2_=milk powder, X_3_=sweet apricot kernel powder, TPC=total phenolic content, DPPH=2,2-diphenyl-1-picrylhydrazyl free radical scavenging activity, ABTS=2,2'-azino-bis(3-ethylbenzthiazoline-6-sulphonic acid) radical scavenging activity

In general, the optimised composition of the yoghurt formulation showed a sensory acceptability and met the requirements of panellists in terms of organoleptic properties and physicochemical quality. The statistical models used were significant as they accounted for all linear effects and interactions between the ingredients of the enriched yoghurt with sweet apricot kernels as an ingredient while remaining acceptable to consumers.

### Interpretation of contour plots

**Fig. 1:**
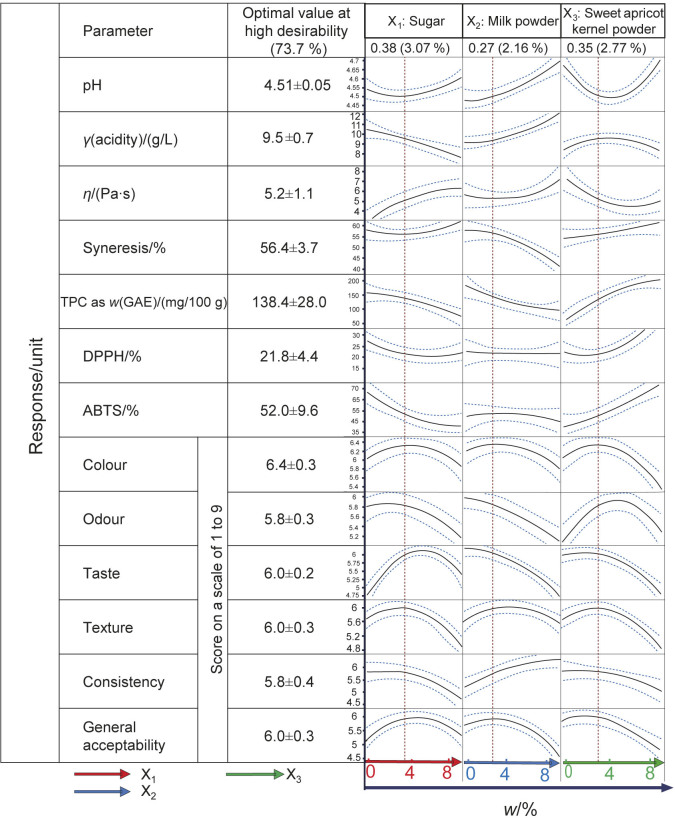
Variation in the physicochemical, sensory and antioxidant properties of yoghurt formulations in response to the mass fractions of sugar (X_1_), milk powder (X_2_) and sweet apricot kernel powder (X_3_)

The coded values of optimal ingredient proportions observed in this study are 0.384 sugar, 0.270 milk powder and 0.346 sweet apricot kernel powder. These values correspond to the real mass fractions of 3.1 % sugar, 2.2 % milk powder and 2.8 % sweet apricot kernel powder. The responses for this optimal combination in high desirability at 73.7 % are 4.51±0.05 for pH, (9.5±0.7) g/L for acidity, (5.2±1.1) Pa·s for viscosity and (56.4±3.7) % for syneresis. The values for ABTS and DPPH radical scavenging activities were (52.0±9.6) and (21.8±4.4) %, respectively. The determined TPC value, expressed as GAE, was (138.4±28.0) mg/100 g. The maximum score on the hedonic scale for the sensory characteristics of colour, odour, taste, texture and consistency was 6.4±0.3, 5.8±0.3, 6.0±0.2, 6.0±0.3 and 5.8±0.4, respectively (Fig. 1).

Regarding the effect of adding sweet apricot kernel powder on TPC, DPPH, ABTS, syneresis, viscosity and acidity, a highly significant increase was observed (p≤0.0001). However, the addition of sweet apricot kernel powder decreased the pH and the interaction between the apricot kernel powder and sugar and/or milk powder exerted a negative linear effect on viscosity and pH (Fig. 1 and Table 5).

Barakat and Hassan (*49*) observed a significant (p<0.05) decrease in pH and an increase in acidity when pumpkin pulp was added to yoghurt. The addition of pumpkin alone increased the viscosity, which was attributed to the acidity and the available carbohydrates and fibre in the pumpkin pulp. This in turn improved the network structure of the stirred yoghurt curd and slightly increased the viscosity. Similarly, Abou-Zeid (*50*) reported that the addition of fibre accelerated the acidification of the yoghurt and that most of the enriched yoghurts also showed an increase in their apparent viscosity.

Therefore, the increase in viscosity and acidity in the present study, as well as the decrease in pH of the produced yoghurts, is probably due to the composition of the sweet apricot kernels and is an indicator of positive bacterial growth. Moreover, the negative effect of X_2_×X_3_ interaction on viscosity can be explained by the presence and dispersion of apricot kernel skin particles in the formulated yoghurt, resulting in gel breakage that occurs between milk protein, carbohydrates and fibre.

Lee *et al.* (*51*) reported that the addition of ginseng extracts provides nutritional components that promote the growth of lactic acid bacteria, resulting in a more rapid increase in the number of viable bacteria, which lowers the pH of yoghurt by converting lactose to lactic acid. Moreover, Sansawat *et al.* (*52*) reported that the growth of lactic acid bacteria during yoghurt fermentation is responsible for the production of exopolysaccharides, which are directly proportional to a higher viscosity. Additionally, yoghurt viscosity is affected by the number and strength of the bonds between the casein micelles, which form casein aggregates from an isoelectric pH<4.9, with maximum gel stiffness at pH=4.6 (*52*).

According to Elkot *et al.* (*14*), the addition of detoxified and delipidated apricot kernel powder increases the viscosity values in yoghurt. This increase may be attributed to the higher total solid and fibre content of the powder. In addition, the powder is characterised by its high water-holding capacity, which influences the aggregation of the casein network in the yoghurt through electrostatic interactions and contributes to the overall resistance of the yoghurt. Soukoulis *et al.* (*45*) observed that skimmed yoghurts were firmer than yoghurts made from whole milk, and they reported that the increase in viscosity of fat-free yoghurts can be attributed to the higher protein content of skimmed milk than of whole milk. Similarly, Brauss *et al.* (*53*) found that yoghurts with higher fat content had a higher viscosity and smaller particle size than yoghurts with lower fat content.

The addition of sweet apricot kernel powder has a significant positive effect on the composition of TPC and antioxidant activity (DPPH and ABTS) (Fig. 1). The yoghurt formulation with 3.1 % sugar, 2.2 % milk powder and 2.8 % sweet apricot kernel powder was the optimal point of the experimental design, with p-value=0.01, indicating a satisfactory optimisation of the multiple responses. The formulated yoghurt had a total phenolic content, expressed as GAE, of (138.5±19.8) mg/100 g and scavenging activities of DPPH and ABTS radicals were 21.8 and 52.0 %, respectively.

The addition of a high amount of sweet apricot kernel powder increased the organoleptic characteristics to an optimal point, which was influenced by the interactions with other ingredients (sugar and milk powder) and resulted in higher acceptance scores. In particular, the addition of sweet apricot kernel powder alone had a significant effect on colour, odour, taste, texture and consistency (p*<*0.0001) (Fig. 1). However, the effect of the interaction with sugar significantly increased the acceptability of taste, colour, odour and texture up to a certain point, beyond which the acceptability by the panel decreased (p*<*0.05).

Moreover, the taste score was influenced by sugar and its interaction with the sweet apricot kernel powder, as well as the lack of appreciation for larger quantities of sweet apricot kernel powder. This can be attributed to the presence of tannins in the apricot kernels, which can contribute to an astringent taste and potentially affect the growth of lactic acid bacteria. Therefore, this can have an impact on the texture, viscosity, taste and odour of the yoghurt.

It is also important to note that excessive sugar addition can result in an overly sweet taste and an overly thick yoghurt. The significant impact of sugar on the texture of yoghurt is worth considering. Sugar is usually added to yoghurt to balance its natural acidity and improve texture. When sugar is added, it forms hydrogen bonds with proteins and attracts water, thereby increasing the viscosity. It can also help form a gel so that the yoghurt retains its shape and consistency.

Recent studies, such as that by Zadeh *et al.* (*54*), have shown that the addition of tannins to yoghurt does not significantly affect the number of lactic acid bacteria, but may influence consumer acceptability. The preference for tannins varies according to their origin, with a higher preference observed for tannins from quebracho wood. In addition, Ibrahim *et al.* (*55*) have shown that the use of tannin-free pomegranate peels can improve the viability of probiotic cultures in yoghurt (*54*). Further studies by Lee *et al.* (*51*) and Barakat and Hassan (*49*) have shown that the addition of ginseng or pumpkin pulp extracts to yoghurt can affect sensory properties such as colour, taste and overall acceptability.

A new product warrants a detailed sensory study combining descriptive and consumer characteristics, in particular using check-all-that-apply (CATA), temporal dominance of sensations (TDS) and flash profile (FP) methods to better understand the optimised formula product and clarify consumer preferences (*56*, *57*). Additionally, studies on the cultural acceptability of the product at retail are necessary to identify the specific aroma profiles resulting from the interactions, with a parallel focus on understanding the cultural acceptability of such products (*47*).

In general, new or unfamiliar food products are often rejected by consumers and tend to receive lower ratings for all sensory attributes (*47*). However, this does not diminish the interest in the inclusion of plant matrices such as fruit seeds as a source of probiotics for lactic acid fermentation and natural antioxidants. For example, Rosa *et al.* (*57*) reported that the addition of prebiotics to dairy products is an excellent opportunity for the dairy industry, contributing to product diversification and in line with the current trend towards functional foods. While this is feasible from a nutritional and commercial point of view, the physicochemical and sensory properties of the product can be maintained or improved (*57*).

### Validation of models

The optimal region was determined by setting the targets such as maximum viscosity and syneresis, minimum acidity, pH in the range of 4.2–4.6, maximum TPC and antioxidant activity, maximum score on the hedonic scale for sensory properties including colour, odour, taste, texture and consistency.

To validate the predicted optimal formula, an experiment with the optimised formulation was carried out in triplicate. It was found that there was no significant difference (p>0.05) between the observed and predicted responses, which indicates the appropriateness of the optimisation process and confirms the validation of the models. The expert panel rated the yoghurt optimised in this study as 'slightly pleasant' with 6 points on a 9-point hedonic scale.

## CONCLUSIONS

In conclusion, the simplex-centroid mixture design successfully predicted the optimal formulation of yoghurt using sweet apricot kernel powder, sugar and milk powder. The addition of sweet apricot kernel powder had a significant positive effect on several parameters, including total phenolic content, antioxidant activity, syneresis, viscosity and acidity. The addition of sugar and milk powder influenced the taste, texture and consistency of the yoghurt. The optimised formulation consisted of 3.1 % sugar, 2.2 % milk powder and 2.8 % sweet apricot kernel powder. These results confirmed the possibility of using sweet apricot kernel powder as an ingredient in yoghurt production, while minimising the amount of sugar and milk powder. In addition, they provide guidance for future efforts in large-scale yoghurt formulation and processing conditions with the aim of achieving a more favourable sensory profile and improving the acceptance of their products by a wider audience.
